# Risk Factors for Refractory Diabetic Macular Oedema after Sub-Tenon's Capsule Triamcinolone Acetonide Injection

**DOI:** 10.1155/2015/195737

**Published:** 2015-09-20

**Authors:** Toshiyuki Oshitari, Yuta Kitamura, Sakiko Nonomura, Miyuki Arai, Yoko Takatsuna, Eiju Sato, Takayuki Baba, Shuichi Yamamoto

**Affiliations:** Department of Ophthalmology and Visual Science, Chiba University Graduate School of Medicine, Inohana 1-8-1, Chuo-ku, Chiba Prefecture, Chiba 260-8670, Japan

## Abstract

The purpose of this study is to identify the risk factors for a recurrence or persistence of diabetic macular oedema (DME) after a sub-Tenon's capsule triamcinolone acetonide (STTA) injection. The medical records of 124 patients (124 eyes) treated by STTA were reviewed. The age, sex, HbA1c level, best-corrected visual acuity, central macular thickness, insulin use, pioglitazone use, systemic hypertension, serous retinal detachment, proteinuria, panretinal photocoagulation, microaneurysm photocoagulation (MAPC), subthreshold micropulse diode laser photocoagulation (SMDLP), cataract surgery, and history of vitrectomy were examined by logistic regression analysis. Procedures of MAPC and SMDLP were significantly associated with DME treated with STTA (*P* = 0.0315, *P* = 0.04, resp.). However, a history of vitrectomy was found to have significantly fewer recurrences or persistent DME after STTA (*P* = 0.0464). In conclusion, patients who required combined MAPC or SMDLP with a STTA injection had significantly higher refractoriness to STTA, but postvitrectomy may prevent the recurrence or persistence of DME after STTA injection.

## 1. Introduction

Diabetic macular oedema (DME) is one of the main causes for reduced visual acuity in patients with diabetes [1]. A recent meta-analysis examined the prevalence of DME in 22,896 diabetic patients and found that 6.81% of diabetic patients had DME [2]. Several prospective, randomized studies showed that intravitreal injections of antivascular endothelial growth factor (VEGF) drugs were effective in reducing macular thickness and improving the visual acuity in patients with DME [3–6]. However, the injections had to be repeated which increased the risk of postintravitreal anti-VEGF endophthalmitis and the medical expenses. For example, in the pooled analysis of the RESOLVE and the RESTORE studies, the incidence of endophthalmitis was 1.4% at 1 year for multiple injections [3].

Growing evidence indicates an association between the intraocular inflammation induced by diabetic stress and the development and progression of DME [7]. Several basic studies demonstrated that steroids upregulate the tight junction proteins, occludin and ZO-1, tighten the retinal blood barrier [8], and reduce the expression of VEGF [9, 10]. Thus, posterior sub-Tenon's capsule injection of triamcinolone acetonide (STTA) [11] and intravitreal injections of triamcinolone acetonide (IVTA) [12] have been used to treat DME.

IVTA has a higher risk of endophthalmitis and elevation of the intraocular pressure than STTA. The Japanese survey of triamcinolone acetonide for ocular diseases reported that the incidence of endophthalmitis by IVTA and STTA was 0.12% and 0.008%, respectively, and that the incidence of glaucoma requiring filtration surgery after IVTA and STTA was 0.56% and 0.26%, respectively [13]. Thus, STTA has a lower risk of endophthalmitis and secondary glaucoma than IVTA.

Our recent study indicated that the short-term effect of STTA for DME is comparable to that of pars plana vitrectomy [14]. However, the benefits of steroid therapies were no longer evident at 6 months [15]. Thus, repeated injections or additional treatments such as laser photocoagulation are usually required for the treatment of DME.

The main purpose of this study was to identify the risk factors that led to a recurrence or persistence of DME after a STTA injection.

## 2. Methods

The medical records of 124 eyes of 124 patients with DME that had STTA between January 2010 and July 2011 at the Chiba University Hospital were reviewed. All of the procedures conformed to the tenets of the World Medical Association Declaration of Helsinki. A signed informed consent was obtained from all patients regarding the procedures to be performed, and approval for this study was obtained from the Institutional Review Board of Chiba University Graduate School of Medicine.

The definition of a recurrence of DME in this study was an eye which initially had a ≧30% decrease of central macular thickness (CMT) compared with the baseline within 1 year after STTA but then increased by ≧30%. The definition of a persistence of DME was an eye which had <30% decrease of the CMT within 1 year after STTA. Seventy-four patients (59.7%; 42 men, 32 women) had a persistent DME or a recurrence within 1 year. In the eyes with a recurrence, the mean interval until the recurrence was 7.7 ± 3.5 months.

The possible risk factors for a recurrence or persistence of a DME after STTA were the age, sex, glycohemoglobin A1c (HbA1c) level, best-corrected visual acuity (BCVA), CMT, insulin use, pioglitazone use, systemic hypertension, serous retinal detachment (SRD), proteinuria, panretinal photocoagulation (PRP), microaneurysm photocoagulation (MAPC), subthreshold micropulse diode laser photocoagulation (SMDLP), cataract surgery, and history of vitrectomy. All possible risk factors for recurrence or persistence of DME after STTA were determined by logistic regression analysis. A* P *< 0.05 was considered significant.

## 3. Results

The baselines clinical characteristics of the 124 patients with DME are shown in Table 1. The DME of 50 eyes (40.3%) was improved after the STTA injection without any additional treatments, 52 (41.9%) had a recurrence of DME, and 22 eyes (17.7%) had a persistence of the DME after STTA injection. All of the results of multiple logistic regression analyses of the risk factors for a recurrence or persistence of DME after the STTA injection are shown in Table 2.

At the time of treatment, the mean age was 60.3 ± 13.1 years, mean HbA1c was 6.7 ± 1.2%, mean BCVA was 0.6 ±  0.4log⁡MAR units, and mean CMT was 539.2 ± 156.7 *µ*m. Thirty-eight patients (30.6%) used insulin, 10 used pioglitazone (8.1%), 55 (44.4%) had hypertension, and 47 (37.9%) had proteinuria. Fifty-five patients (44.4%) underwent PRP and 25 patients (20.2%) underwent cataract surgery. None of these factors was found to be significant risk factors (Tables 1 and 2).

Forty-nine patients (39.5%) underwent MAPC and 13 patients (10.5%) underwent SMDLP combined with the STTA injection. These procedures were found to be significantly associated with a persistence or recurrence of the DME (*P* = 0.0315,* P* = 0.04, resp.; Table 2). On the other hand, 17 patients (13.7%) with a history of PPV had significantly fewer recurrences or persistence of DME after STTA injection (*P* = 0.0464; Table 2).

## 4. Discussion

The results indicated that 40% of patients with DME were successfully treated with a single STTA injection without any additional treatments for at least 1 year. On the other hand, 60% of DME patients had a recurrence or persistence of DME after a single STTA injection and repeated STTA injections or other treatments were required.

The specific indications for MAPC and SMDLP were not determined but the patients with microaneurysms at the posterior pole underwent STTA combined with MAPC immediately or within one month after the STTA injection. The effect of the STTA injection for DME is rapid, and our results indicate that the CMT was significantly reduced 1 month after the STTA injection [14]. In our hospital, SMDLP was determined to be a better treatment than grid laser photocoagulation for DME [16]. Thus, the refractory DME patients without microaneurysms underwent SMDLP and not grid laser photocoagulation as additional treatment after STTA. Basically, both MAPC and SMDLP tended to be performed for patients with mild to moderate DME with CMT >500 *μ*m in our hospital. Therefore, patients with severe DME with CMT <500 *μ*m underwent STTA first, followed by undergoing MAPC or SMDLP.

The results of the logistic regression analysis indicated that both MAPC and SMDLP were risk factors for a recurrence or persistence of DME because these patients tended to have refractory DME and needed to undergo additional treatments. Although patients who underwent MAPC had an enough population size, smaller number of patients underwent SMDLP compared to MAPC. Thus, the result of the logistic regression analysis for SMDLP should be interpreted with caution.

Recently Ribeiro et al. suggested that a high microaneurysm turnover rate (sum of the microaneurysm formation and disappearance rates) was a higher risk for developing clinical significant macular oedema (CSME) over a 2-year period [17]. Haritoglou et al. demonstrated that high microaneurysm formation rate was a predictive marker for progression to CSME for a period of up to 5 years [18]. Taken together, microaneurysm formation is probably a sign of severe diabetic stress including oxidative stress in the macula of diabetic patients, and the requirement of MAPC combined with STTA injections may be necessary to treat the DME. However, these patients may increase diabetic stress including oxidative stress in the macula; further pathological changes such as Müller cell swelling, retinal pigment epithelium (RPE) dysfunction, or blood-retinal barrier dysfunction may be accompanied. Such pathological changes may cause refractoriness of DME after STTA with MAPC.

Although the precise mechanism of the effect of SMDLP is unclear, SMDLP may stimulate and activate RPE and improve to draw out the excessive fluid in the retina. But steroid can affect the function of RPE in diabetic patients. Thus, STTA may not be fitted with SMDLP because of exacerbating RPE function.

Vitrectomized eyes had a significantly lower risk for recurrence or persistence of DME after the STTA injection. One possible reason for this is that vitrectomized eyes have no vitreomacular traction. Another possible reason is that, in vitrectomized eyes, pathological cytokines, such as VEGF or IL-6, can easily diffuse and are not in contact with the macula for a long period. Thus, a STTA injection may be one of the options for treatment of DME developing in vitrectomized eyes but a careful management of the steroid response is needed because glaucoma infiltration surgeries are difficult to perform in vitrectomized eyes.

The HbA1c level is known to be a major risk factor for developing DME [19, 20]. However, in this study, the HbA1c level was not found to be a significant risk factor for recurrence or persistence of DME after a STTA injection from the logistic regression analysis. We have classified the grades of DM control as good control group (HbA1c < 6.5%), moderated control group (6.5% ≦ HbA1c ≦ 8.0%), and poor control group (8.0% < HbA1c) and reevaluated whether the DM control was risk factors for recurrence or persistence of DME after STTA injection. But the DM controls have not been identified as risk factors for recurrence or persistence of DME after STTA injection (*P* = 0.2203). However, 80% of the patients with HbA1c levels >9.0% had a recurrence or persistence of the DME after the STTA injection. Thus, a poor glycemic control seems to increase a risk of recurrence or persistence of DME after STTA injection.

The results of two large cohort studies indicated that glitazone is a risk factor for developing DME [21, 22], and we have reported the first case of DME after pioglitazone use in Japan [23]. However, in this study, pioglitazone was not found to be a risk factor for recurrence or persistence of DME after a STTA injection.

From this study, we tentatively suggest the indication of STTA in the clinical practice. STTA may be selected for treatment of DME without MAs or DME after vitrectomy. DME with MAs may be treated with intravitreal injection of anti-VEGF antibodies. SMDLP may not be fitted with STTA. Further studies are needed to evaluate the additive effect of intravitreal injection of anti-VEGF antibodies with SMDLP for patients with DME.

## 5. Conclusions

Although this study has a limitation because of its retrospective nature, patients who needed to have combined MAPC and SMDLP with a STTA injection had significantly higher refractoriness to DME. However, vitrectomized eyes may reduce the incidence of recurrence or persistent DME after a STTA injection. Additional prospective studies are needed to confirm the risk factors for a recurrence or persistent DME after a STTA injection.

## Figures and Tables

**Table 1:** Baseline clinical characteristics of 124 patients with DME.

Factors	Cases (124 eyes)
Sex, men : women (*n*)	71 : 53
Mean age (y.o.)	60.3 ± 13.1
Mean HbA1c (%)	6.7 ± 1.2
Hypertension, + : − (*n*)	55 : 69
Insulin use, + : − (*n*)	38 : 86
Pioglitazone use, + : − (*n*)	10 : 114
Proteinuria, + : − (*n*)	47 : 77
Mean BCVA (logMAR)	0.6 ± 0.4
Mean CMT (*μ*m)	539.2 ± 156.7
SRD, + : − (*n*)	33 : 91
MAPC, + : − (*n*)	49 : 75
SMDLP, + : − (*n*)	13 : 111
PRP, + : − (*n*)	55 : 69
Vitrectomy, + : − (*n*)	17 : 107
Cataract surgery, + : − (*n*)	25 : 99

BCVA: best-corrected visual acuity, CMT: central macular thickness, SRD: serous retinal detachment, MAPC: microaneurysm photocoagulation, SMDLP: subthreshold micropulse diode laser photocoagulation, and PRP: panretinal photocoagulation.

**Table 2:** Multiple logistic regression analysis of a recurrence and/or persistence of DME after STTA.

Factors	Recurrence (+)	Recurrence (−)	Odds ratio	95% CI	*P* value
Sex, men : women (*n*)	42 : 32	29 : 21	0.718	0.034–0.973	0.4846
Mean age (y.o.)	61.6 ± 13.0	58.5 ± 13.2	1.012	0.978–1.049	0.4883
Mean HbA1c (%)	6.83 ± 1.32	6.59 ± 1.09	1.239	0.833–1.843	0.2906
Hypertension, + : −	38 : 36	17 : 33	1.926	0.760–4.879	0.1669
Insulin use, + : − (*n*)	24 : 50	14 : 36	1.367	0.525–3.562	0.5218
Pioglitazone use, + : − (*n*)	8 : 66	2 : 48	2.416	0.373–15.628	0.3544
Proteinuria, + : − (*n*)	30 : 44	17 : 33	1.103	0.420–2.894	0.8422
Mean BCVA (logMAR)	0.61 ± 0.37	0.52 ± 0.33	2.054	0.523–8.065	0.3022
Mean CMT (*μ*m)	557.2 ± 143.7	512.8 ± 172.2	1.002	0.999–1.005	0.1800
SRD, + : − (*n*)	22 : 52	11 : 39	1.012	0.370–2.769	0.9821
MAPC, + : − (*n*)	35 : 39	14 : 36	2.566	1.087–6.058	0.0315
SMDLP, + : − (*n*)	11 : 63	2 : 48	7.772	1.098–55.004	0.0400
PRP, + : − (*n*)	33 : 41	22 : 28	0.951	0.390–2.321	0.9124
Vitrectomy, + : − (*n*)	7 : 67	10 : 40	0.182	0.034–0.973	0.0464
Cataract surgery, + : − (*n*)	15 : 59	10 : 40	1.355	0.336–5.460	0.6691

BCVA: best-corrected visual acuity, CMT: central macular thickness, SRD: serous retinal detachment, MAPC: microaneurysm photocoagulation, SMDLP: subthreshold micropulse diode laser photocoagulation, and PRP: panretinal photocoagulation.
